# Effects of autumn diurnal freeze–thaw cycles on soil bacteria and greenhouse gases in the permafrost regions

**DOI:** 10.3389/fmicb.2022.1056953

**Published:** 2022-12-01

**Authors:** Zhenying Lv, Yuzheng Gu, Shengyun Chen, Jianwei Chen, Yinglan Jia

**Affiliations:** ^1^Cryosphere and Eco-Environment Research Station of Shule River Headwaters, State Key Laboratory of Cryospheric Science, Northwest Institute of Eco-Environment and Resources, Chinese Academy of Sciences, Lanzhou, China; ^2^Key Laboratory of Petroleum Resources, Gansu Province, Lanzhou, China; ^3^College of Grassland Agriculture, Northwest A&F University, Yangling, China; ^4^State Key Laboratory of Grassland and Agro-Ecosystems, College of Ecology, Lanzhou University, Lanzhou, China; ^5^Long-Term National Scientific Research Base of the Qilian Mountain National Park, Xining, China; ^6^BGI Research-Qingdao, BGI, Qingdao, China; ^7^University of Chinese Academy of Sciences, Beijing, China

**Keywords:** diurnal freeze–thaw cycles, soil bacteria, greenhouse gas fluxes, permafrost regions, Qinghai-Tibet Plateau

## Abstract

Understanding the impacts of diurnal freeze–thaw cycles (DFTCs) on soil microorganisms and greenhouse gas emissions is crucial for assessing soil carbon and nitrogen cycles in the alpine ecosystems. However, relevant studies in the permafrost regions in the Qinghai-Tibet Plateau (QTP) are still lacking. In this study, we used high-throughput pyrosequencing and static chamber-gas chromatogram to study the changes in topsoil bacteria and fluxes of greenhouse gases, including carbon dioxide (CO_2_), methane (CH_4_), and nitrous oxide (N_2_O), during autumn DFTCs in the permafrost regions of the Shule River headwaters on the western part of Qilian Mountains, northeast margin of the QTP. The results showed that the bacterial communities contained a total of 35 phyla, 88 classes, 128 orders, 153 families, 176 genera, and 113 species. The dominant phyla were *Proteobacteria*, *Acidobacteria*, *Actinobacteria*, *Chloroflexi*, and *Gemmatimonadetes*. Two DFTCs led to a trend of increasing bacterial diversity and significant changes in the relative abundance of 17 known bacteria at the family, genus, and species levels. These were predominantly influenced by soil temperature, water content, and salinity. In addition, CO_2_ flux significantly increased while CH_4_ flux distinctly decreased, and N_2_O flux tended to increase after two DFTCs, with soil bacteria being the primary affecting variable. This study can provide a scientific insight into the impact of climate change on biogeochemical cycles of the QTP.

## Introduction

Soil freeze–thaw cycles are repeated freezing–thawing physical processes that occur due to diurnally or seasonally thermal changes at certain topsoil depths, which are common natural phenomena at high elevation and high latitude regions (Grogan et al., 2004; Chen et al., 2018a), and are sensitive to climate change (Song et al., 2017). Climate warming scenarios are expected to induce an increase in the frequency of freeze–thaw cycles (Kreyling et al., 2008; Gao et al., 2021), especially diurnal freeze–thaw cycles (DFTCs) (Ren et al., 2018). In general, freeze–thaw processes can be divided into seasonal freeze–thaw processes and DFTCs, the former including thawing, completely thawed, freezing, and completely frozen periods (Chen et al., 2020), the latter can be defined as soil temperature (Ts) rising above 0°C at some times of the day and falling below 0°C at other times, mainly during thawing and freezing periods (Yang et al., 2007; Ren et al., 2018). It is estimated that 55% of land surface experiences seasonal freeze–thaw cycles in the Northern Hemisphere (Zhang et al., 2003). The Qinghai-Tibet Plateau (QTP) is the most widely distributed region of high-altitude permafrost in the Northern Hemisphere and is the youngest, highest, and largest plateau in the world, with an average elevation of 4,000 m and an area of approximately 2.5 × 10^6^ km^2^. As one of the main types of soil erosion, freeze–thaw cycles are widely distributed over an area of 149.02 × 10^4^ km^2^, accounting for 62.2% of the total area in the QTP (Wang et al., 2017). Consequently, the alpine permafrost regions undergo more frequent freeze–thaw cycles, particularly the DFTCs during the thawing period in spring and the freezing period in autumn, due to the broad daily temperature range (Yang et al., 2007).

Many studies have reported the direct and indirect effects of freeze–thaw cycles on the soil environment, including physicochemical properties, microorganisms, and greenhouse gases (GHGs) (Wang et al., 2013; Ren et al., 2018; Schostag et al., 2019; Gao et al., 2022). Zhao et al. (2000) found that soil hydrothermal transportation was more dynamic in the active layer of the alpine steppe in the permafrost regions of the central QTP during seasonal freeze–thaw cycles. Recurrent freeze–thaw cycles are phase transitions of soil moisture triggered by fluctuating temperature, leading to a decrease in soil aggregate stability (Oztas and Fayetorbay, 2003; Six et al., 2004), and an increase in soil total porosity (Deng et al., 1999; Xie et al., 2015). Accompanying these changes, soil organic matter is increased by physically destroying microbial cells and aggregates (Grogan et al., 2004; Feng et al., 2007; Yu et al., 2011). These additional substrates can enhance availability, provide a C source for surviving microorganisms, and improve microbial activity, which can then substantially influence the structure and function of the microbial communities (Sharma et al., 2006; Feng et al., 2007; Männistö et al., 2009; Sawicka et al., 2010). However, the different responses of soil microorganisms to freeze–thaw cycles might depend on the research methods employed. For example, Feng et al. (2007) revealed that freeze–thaw cycles did not affect bacteria, whereas fungal biomass was greatly reduced through laboratory simulation. In contrast, Ren et al. (2018) found that autumn DFTCs decreased soil bacteria and methanogens based on an in-situ experiment. Moreover, freeze–thaw cycles might enhance the microbial decomposition of soil organic matter and lead to GHGs emissions (Risk et al., 2013; Wang et al., 2013; Song et al., 2017), resulting in positive climate feedback. Although some laboratory experiments and in-situ experiments have observed increased emissions of CO_2_, CH_4_, and N_2_O during freeze–thaw cycles (Sharma et al., 2006; Tokida et al., 2007; Song et al., 2012; Raz-Yaseef et al., 2017), there were great differences in the amount of greenhouse gas emitted. The main reasons for these differences might be attributed to soil types, physicochemical properties, freeze–thaw patterns, and soil microbial activity (Zhou et al., 2019; Wu et al., 2020).

In recent years, increasing attention has been paid to the response of the soil environment to freeze–thaw cycles, but many studies were mainly conducted using climate chambers in the laboratory (Sharma et al., 2006; Feng et al., 2007; Männistö et al., 2009; Yu et al., 2011; Koponen and Baath, 2016; Han et al., 2018). Although the laboratory experiment had great advantages in revealing the underlying mechanisms of freeze–thaw cycles on soil carbon and nitrogen cycles and supporting model parameterization, some reports were based on single-factor laboratory experiments that could not fully replicate the realistic field conditions (Henry, 2007; Chen et al., 2018b); Consequently, it was difficult to reflect the true changing regularity. To date, there is little knowledge about the effects of freeze–thaw cycles on soil bacteria and greenhouse gas fluxes in the permafrost regions of the QTP, either through laboratory experiment or field observation. To explore the effects of autumn DFTCs on soil bacteria and GHGs, we observed topsoil bacteria of the active layer and surface greenhouse gas fluxes before and after DFTCs in the permafrost regions of the Shule River headwaters on the western part of Qilian Mountains, northeast margin of the QTP. Our objectives were to: (1) investigate composition and diversity of topsoil bacterial communities in the permafrost regions during the onset of the autumn freezing period; (2) explore the responses of soil bacterial communities and greenhouse gas fluxes to DFTCs; and (3) discuss their influencing variables and the relationship between them. This study will enrich the scientific knowledge about the effect of freeze–thaw cycles on soil microorganisms, carbon and nitrogen cycling, and ecosystem function in the alpine permafrost regions under a future climate warming scenario.

## Materials and methods

### Site description

**Figure 1:**
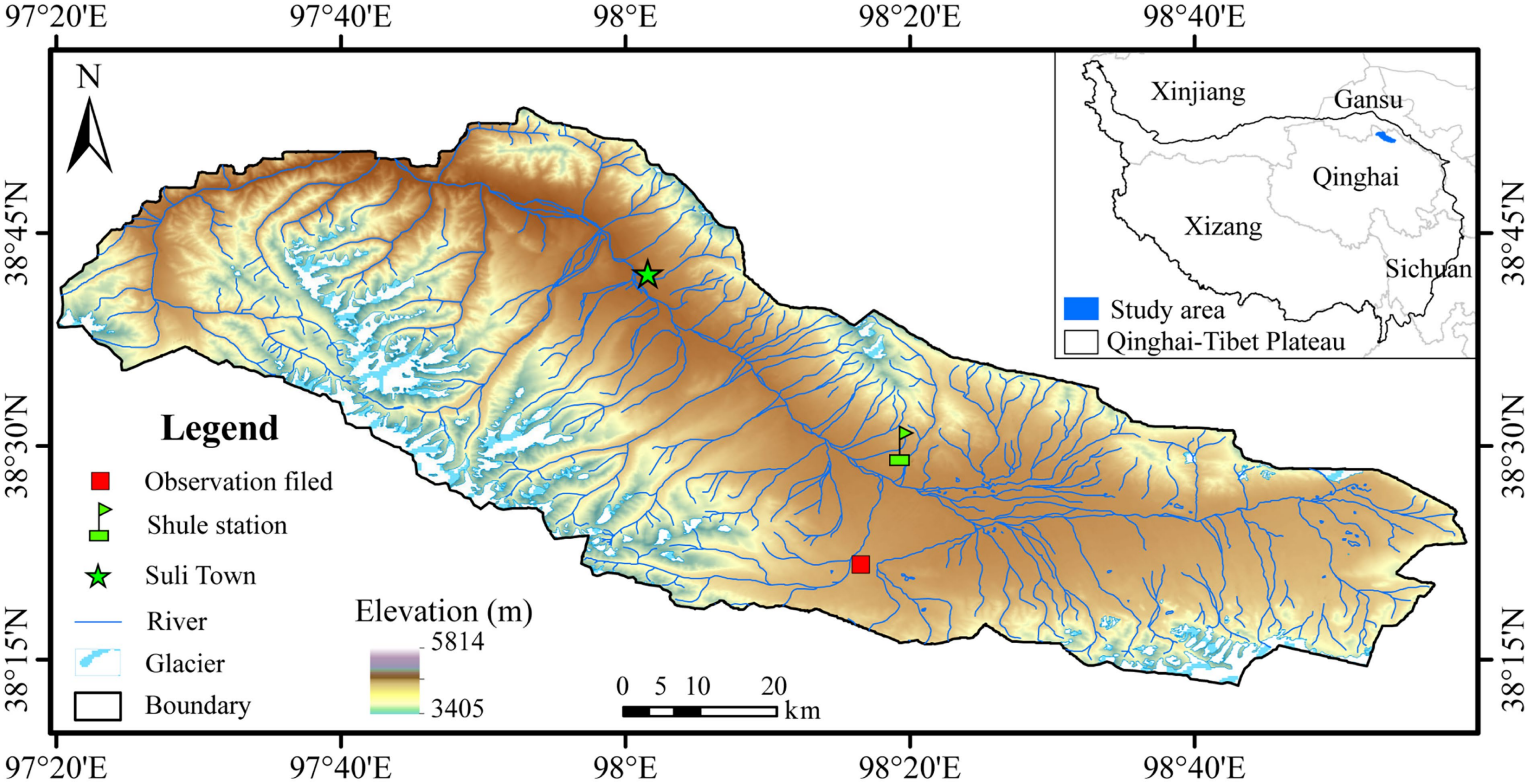
Location of the study area and sampling site.

The study site was located in an observational field of the alpine meadow ecosystem (98^o^16ʹ14″ E, 38^o^21ʹ17″ N, *Alt.*: 4014 m) (Figure 1) in the permafrost regions of the Shule River headwaters, the western part of the Qilian Mountains, northeastern margin of the QTP, China, which has a continental arid desert climate (Chen et al., 2012). The region has a continental arid desert climate. Based on the meteorological monitoring data, the average annual air temperature (Ta) and precipitation were approximately −3.5°C and 359 mm, respectively. The coldest and warmest months were January and August, with the mean Ta of −16.6°C and 8.9°C, respectively. The vegetation type belongs to the alpine meadow with a community coverage of approximately 42%, and the dominant plants are *Kobresia pygmaea* and *Kobresia humilis* (Chen et al., 2012). According to the Chinese Soil Classification System, the soil type is cold calcic soil (Liu et al., 2012a). The permafrost type is divided into sub-stable mountain permafrost, with an active layer approximately 2.3 m thick (Chen et al., 2012).

The number of diurnal freeze–thaw days was calculated by those days with the diurnal maximum Ts being >0.0°C and the diurnal minimum Ts being ≤0.0°C (Chen et al., 2018a). Therefore, daily maximum Ta and Ts, daily minimum Ta and Ts, and daily Ta and Ts were registered from October 1–6, 2011. The first sampling time on October 1 was assumed to be the period before DFTCs (pre-DFTCs). However, if DFTCs occurred until the second sampling time on October 6, this time was defined as the period after DFTCs (post-DFTCs).

### Sample collection and measurement

Three squares (50 × 50 cm) were randomly chosen in the observational field on October 1, 2011. After aboveground vegetation in each square was harvested, soil samples of the active layer at a depth of 0–10 cm were collected by combining five soil cores (4.8 cm in diameter) in an X-shaped pattern and then divided into three parts. One part for bacterial detection was stored in a freezer box and transported to the laboratory. Another part was stored in a cold storage box to analyze soil oxidation–reduction potential (Eh), pH, soil microbial biomass carbon (SMBC), and microbial biomass nitrogen (SMBN). The last part was air-dried under natural conditions to test for variables including soil particle size, specific gravity (SG), soil organic carbon (SOC), and total nitrogen (TN). On October 6, using the same approach, soil samples were collectedand analyzed for soil microbes and other variables, excluding vegetation biomass and soil particle size. Soil bulk density (BD) was measured using a cutting ring (volume of 100 g·cm^−3^) on October 1 and 6. Nearby each square, at least 2 m away, greenhouse gas samples were acquired using the static-chamber method (Liu et al., 2012b; Yu et al., 2013) and were obtained using 100 ml syringe each time between October 1 and 6. Ta and relative humidity (RH) at 1.5 m high, Ts, soil water content (SWC), and soil salinity (SS) at 0–10 cm depth (at memorized intervals of 10 min) from October 1–6 were automatically measured using an HMP155A Air Temperature probe (Vaisala, Finland) and Hydra Probe II soil sensor (Stevens, United States) connected to a CR1000X datalogger (Campbell Scientific, United States), respectively.

The SG and soil particle size were determined using the pycnometer methods (ISSCAS Institute of Soil Science, Chinese Academy of Sciences, 1978) and wet sieving (Lu et al., 2022), respectively. Soil porosity (Por) was calculated using the equation: $\left(\mathrm{Por}\left(\%\right)=\left(1-\frac{\mathrm{BD}}{\mathrm{SG}}\right)\times 100\%\right)$ . SOC and TN were analyzed by dichromate oxidation using the Walkley-Black procedure (Nelson and Sommers, 1983) and micro-Kjeldahl procedures (ISSCAS Institute of Soil Science, Chinese Academy of Sciences, 1978), respectively. The soil pH and Eh were measured using E-301-CF and 501-ORP composite electrodes connected to a PHBJ-260 portable pH meter (INESA, China) with a soil: water ratio of 1:5, respectively. Soil microbial biomass, including SMBC and SMBN, was determined using the Walkley-Black procedure and colorimetric methods after chloroform fumigation and 0.5 mol·L^−1^ potassium sulfate extraction, respectively (Liu et al., 2012b).

The concentrations of GHGs (CO_2_, CH_4_, and N_2_O) were determined using a 7890A gas-chromatography system (Agilent Tec., United States). The calibrated standard gas mixtures of CO_2_ (402.97 ml/m^3^), CH_4_ (2.2457 ml/m^3^), and N_2_O (0.3240 ml/m^3^) after several rounds of calibration were provided by the Chinese Academy of Meteorological Sciences, China Meteorological Administration, and their concentration values were obtained via the international primary standards set by the Central Calibration Laboratory, the World Meteorological Organization (Chen et al., 2018a). Soil greenhouse gas fluxes were calculated using the equation described in our previous study (Liu et al., 2012b).

### DNA extraction, PCR, and pyrosequencing

Genomic DNA was extracted using the PowerSoil^®^ DNA Isolation Kit (MoBio, Laboratories, Carlsbad, CA, United States) following the manufacturer’s instructions. The extracted DNA was checked on a 1% agarose gel and the concentration was determined using a Nanodrop ND-1000 UV–Vis Spectrophotometer (NanoDrop Technologies, United States). The V5–V3 region of the 16S rRNA gene was amplified using the primer pair 907F (5′-CCGTCAATTCMTTTGAGTTT-3′) and 338R (5′-ACTCCTACGGGAGGCAGCAG-3′). The PCR reaction contained 5 μl 10 × PFX buffer, 2 μl MgSO_4_, 2 μl dNTPs, 2 μl of 10 μM each primer, 0.8 μl PFX polymerase, and 1 μl genomic DNA extract. The PCR profile was initial denaturation at 94°C for 3 min, followed by 30 cycles of denaturation at 94°C for 30 s, annealing at 55°C for 30 s, and extension at 70°C for 45 s, followed by a final extension at 70°C for 7 min. The PCR products were purified using the AxyPrep DNA Gel Extraction Kit (AxyPrep) and were quantified by the TBS-380 system. Then, equal amounts of all amplicons were mixed in a single tube. Emulsion PCRs were performed based on the Amplicon Library Preparation Manual and sequencing was performed on a Roche 454 GS FLX Platform.

### Bioinformatic

The raw sequences produced from spanning V5–V3 hypervariable regions were processed using Mothur software (V 1.31.2) to obtain unique reads. These reads were first assigned to samples according to their tags, and then the following criteria were applied: (1) almost a perfect match with the barcode and primer. “Almost a perfect match” means one mismatch/deletion/insertion is allowed in the barcode, idem for the primer (De Filippo et al., 2010); (2) length of at least 200 nucleotides or at most 1,000 nucleotides (barcodes and primers excluded); (3) no more than two undetermined bases (denoted by N); (4) all reads were aligned against the SILVA reference alignment (V102) taking NAST algorithm, sequences that did not align or did not align over the expected region of the alignment were discarded (Pruesse et al., 2007); and (5) if the reads were identified as a chimera with UCHIME arithmetic, then the chimeric sequences should be discarded (Edgar et al., 2011). Based on the quality control presented above, the non-redundant sequences were clustered into operational taxonomic units (OTUs) at 97% sequence identity using USEARCH (V7.0.1090).

### Statistical analysis

All statistical analyses were executed using the R software (V4.1.0), and all figures were generated using the “ggplot2” R package and ArcGIS (V10.5). The prcomp function in the factoextra R package was used to quantify bacterial community compositions pre- and post-DFTCs, and the significant test of difference was inspected using permutational multivariate analysis of variance (PERMANOVA; 999 permutations) with the “vegan” R package. Significant differences in OTUs numbers and the relative abundance of soil bacteria at different taxonomic levels of pre- and post-DFTCs were calculated using the Wilcoxon rank-sum test. One-way analysis of variance (ANOVA) was used to evaluate the significant differences between pre-and post-DFTCs for environmental variables, soil microbial biomass, and greenhouse gas fluxes, respectively. Redundancy analysis between soil bacteria and environmental variables was performed to reflect the relationship between environmental variables and soil bacteria using the “vegan” R package. Shannon, Chao, and Simpson indices (alpha diversity) were calculated using the diversity function in the “vegan” R package. Beta diversity was estimated based on the Bray-Curtis distance. The Pearson correlation analysis and the Mantel test were conducted to establish the correlation between environmental variables and soil bacteria, and their correlation with soil greenhouse gas fluxes was performed using the “vegan” R package. To select the optimal sets of variables that were significantly related to soil greenhouse gas fluxes, forward selection of soil bacteria which varied significantly after two DFTCs and environmental variables was conducted using Ordistep function in the “vegan” R package, respectively. Multivariate regression analysis with the LM function was performed to calculate the contributions of environmental variables and soil bacteria to soil greenhouse gas fluxes.

## Results

### Main features of environmental variables

During the onset of the autumn freezing period from October 1–6, 2011, Ta, Ts, SWC, and SS indicated the tendency of fluctuation reduction, while the daily Ta range gradually increased by 3.08°C, with an increase in daily maximum Ta and a decrease in daily minimum Ta (Supplementary Figures S1A–C). The soil silt and sandy fractions were 46.28% and 41.92%, respectively. The soil texture was classified as loam according to the USDA soil texture classification standard (Miller and White, 1998). The BD, Por, Eh, and pH values were 1.30 g·cm^−3^, 50.16%, 226 mV, and 8.8, respectively. The SOC and TN contents were 19.16 g·kg^−1^ and 1.70 g·kg^−1^, respectively. In addition, diurnal maximum Ts were >0.0°C from October 1–6, but diurnal minimum Ts were ≤0.0°C at 8:10–8:30 a.m. (October 5) and 7:20–11:40 a.m. (October 6) (Supplementary Figure S1A); it was confirmed that two DFTCs occurred during this period. The diurnal means of Ts, SWC, and SS of post-DFTCs were significantly decreased compared to those of pre-DFTCs (*p* < 0.05). RH, Ta, Eh, and pH showed decreasing trends, while SOC and TN exhibited opposite trends (Table 1).

**Table 1:** Differences of environmental variables, soil microbial biomass and greenhouse gases fluxes pre- and post-DFTCs.

	Mean pre-DFTCs (SE)	Mean post-DFTCs (SE)	Differ. (SE)	*p*-Value
Ta (°C)	0.09 (1.40)	−0.10 (1.63)	−0.18 (0.34)	0.834
RH (%)	47.09 (6.01)	37.21 (5.05)	−9.88 (2.31)	0.208
Ts (°C)	1.97 (0.52)	0.47 (0.16)	−1.50 (0.37)	0.016
SWC (%)	30.49 (0.20)	26.27 (1.27)	−4.23 (1.07)	0.003
SS (mg·L^−1^)	149.00 (2.16)	126.90 (5.42)	−22.10 (3.40)	0.003
Eh (mV)	233.33 (4.91)	218.67 (10.37)	−14.67 (7.26)	0.270
pH	8.93 (0.09)	8.77 (0.09)	−0.17 (0.09)	0.252
SOC (g·kg^−1^)	17.79 (7.49)	20.53 (10.19)	2.74 (2.56)	0.827
TN (g·kg^−1^)	1.55 (0.50)	1.84 (0.78)	0.30 (0.28)	0.765
C:N	10.88 (1.03)	10.46 (0.80)	−0.42 (0.24)	0.763
SMBC (mg·kg^−1^)	145.92 (23.65)	465.09 (244.34)	319.16 (257.04)	0.275
SMBN (mg·kg^−1^)	9.62 (1.71)	10.21 (2.95)	0.59 (1.16)	0.872
CO_2_F (mg·m^−2^·h^−1^)	61.961 (1.391)	125.068 (45.758)	63.107 (45.654)	0.049
CH_4_F (mg·m^−2^·h^−1^)	0.002 (0.001)	−0.023 (0.009)	−0.039 (0.008)	0.013
N_2_OF (μg·m^−2^·h^−1^)	8.663 (4.137)	14.030 (3.934)	5.367 (4.775)	0.400

Differ., differences pre- and post-DFTCs; SE, standard error; Ta and RH, air temperature and relative humidity; Ts, SWC and SS, soil temperature, soil water content and soil salinity; Eh, soil oxidation–reduction potential; SOC and TN, soil organic carbon and total nitrogen; SMBC and SMBN, soil microbial biomass carbon and nitrogen; CO_2_F, CH_4_F, and N_2_OF, CO_2_ flux, CH_4_ flux, and N_2_O flux. The below were same.

### Soil bacteria changes and their controlling variables

**Figure 2:**
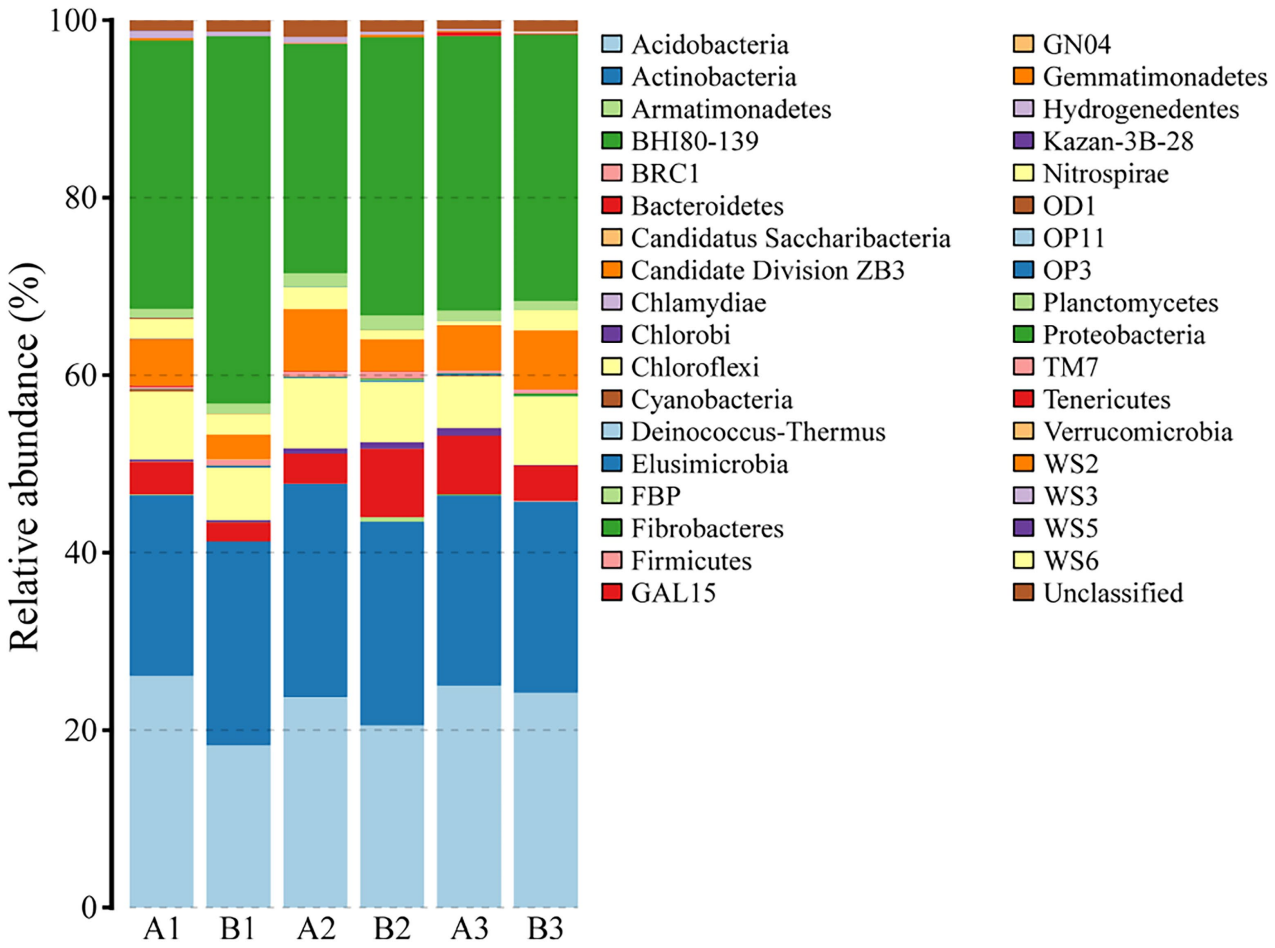
Community composition of soil bacteria at the phylum level. A1, A2, and A3 represented soil samples before DFTCs (pre-DFTCs) on October 1, while B1, B2, and B3 were sampled after DFTCs (post-DFTCs) on October 6, 2011.

A total of 16,054 reads were obtained from all samples and were clustered to 2,237 OTUs corresponding to 35 phyla, 88 classes, 128 orders, 153 families, 176 genera, and 113 species. Figure 2 demonstrated the relative abundance of various bacterial phyla in each sample. Dominant bacterial phyla in six samples were *Proteobacteria*, *Acidobacteria*, *Actinobacteria*, *Chloroflexi*, and *Gemmatimonadetes*, which accounted for 31.6 ± 2.1%, 23.0 ± 1.2%, 22.2 ± 0.5%, 7.0 ± 0.4%, and 5.1 ± 0.7% of the total relative abundance, respectively. Chao, Shannon, and Simpson indices, and beta diversity were 955.3323 ± 14.4753, 5.7640 ± 0.0317, 0.0055 ± 0.0002, and 0.5863 ± 0.0103, respectively.

**Figure 3:**
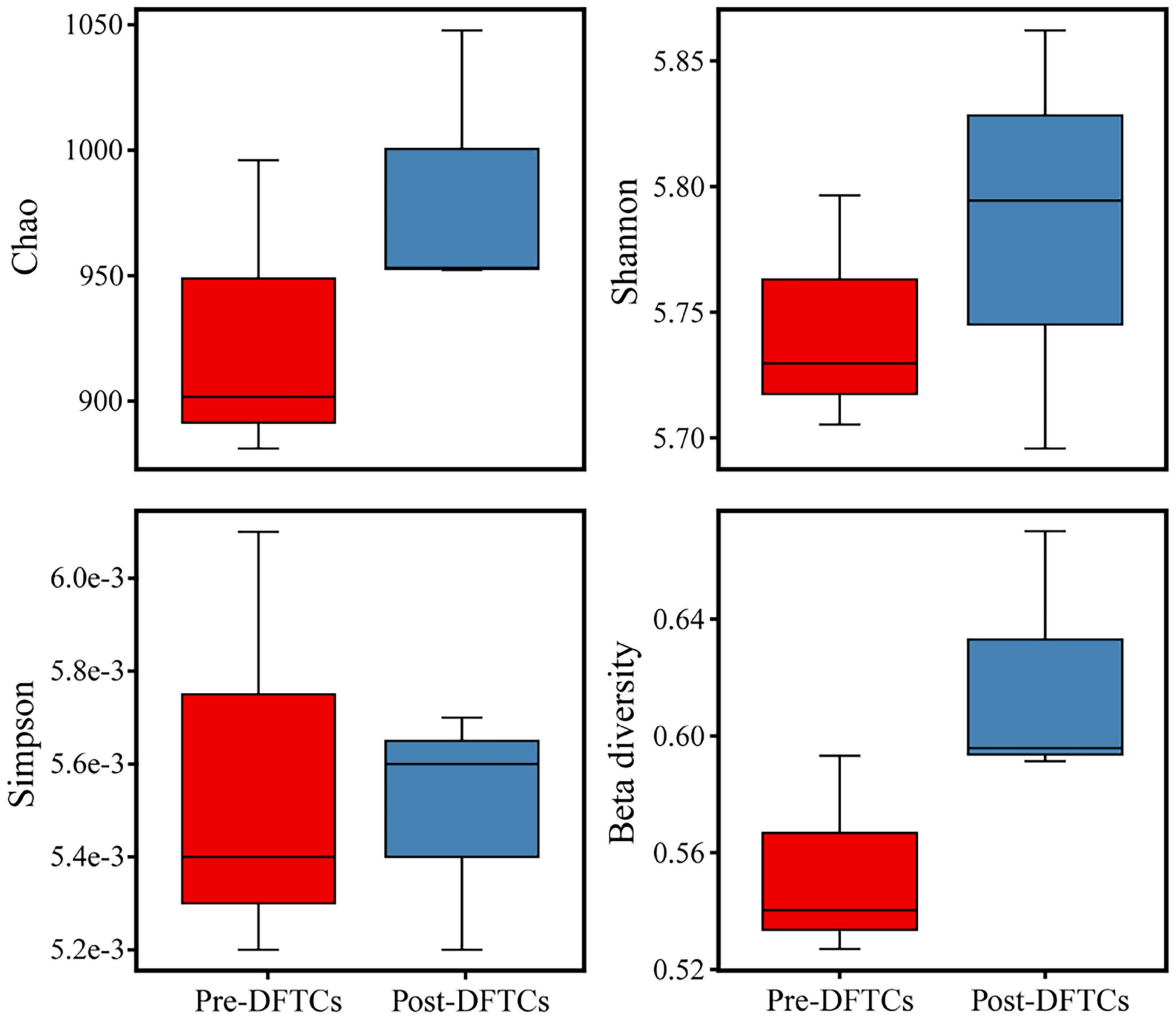
Boxplot of alpha and beta diversities indices for soil bacteria pre- and post-DFTCs.

**Figure 4:**
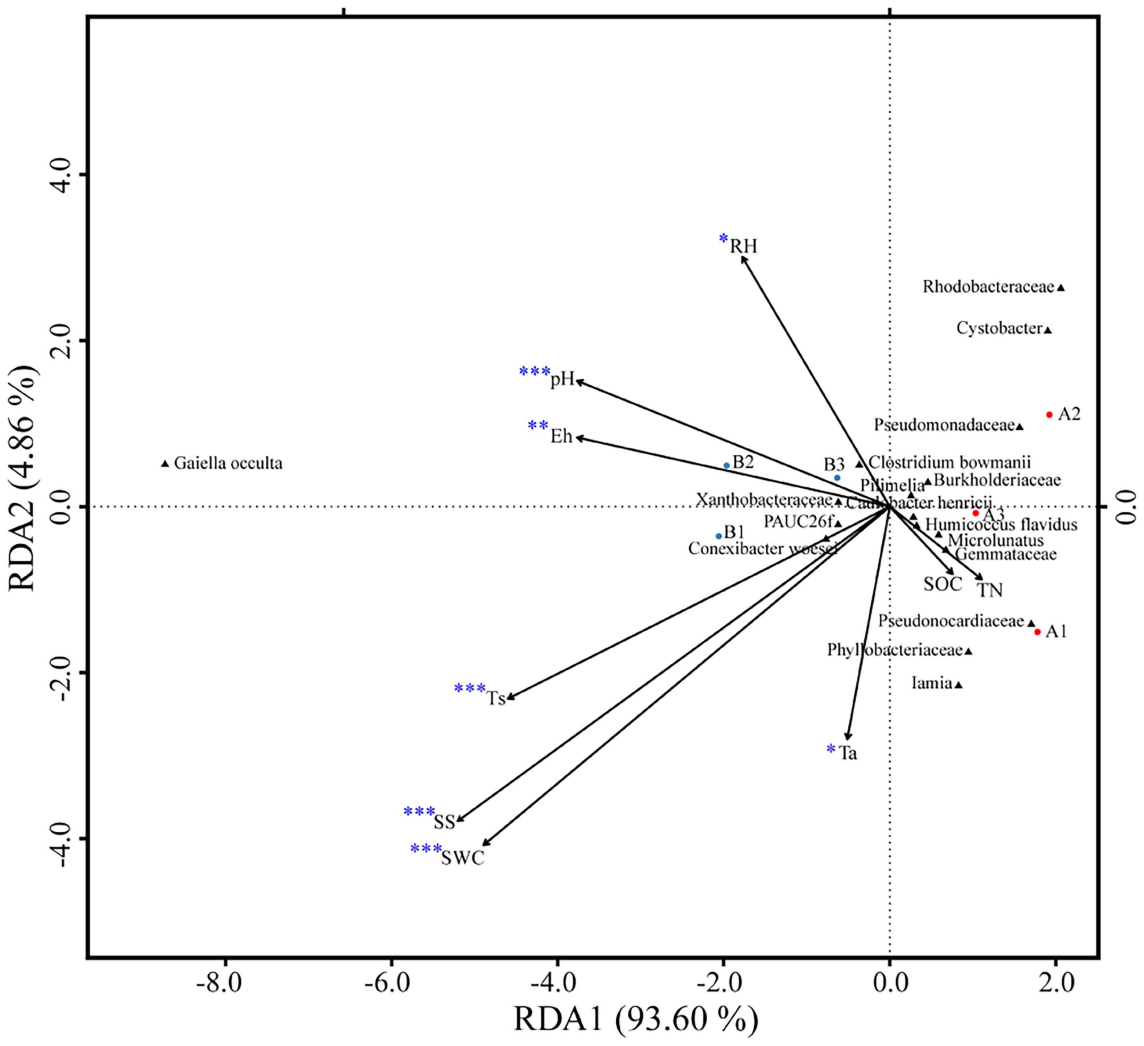
Redundancy analysis of soil bacteria community composition as explained by environmental variables (*p < 0.05; **p < 0.01; and ***p < 0.001).

**Figure 5:**
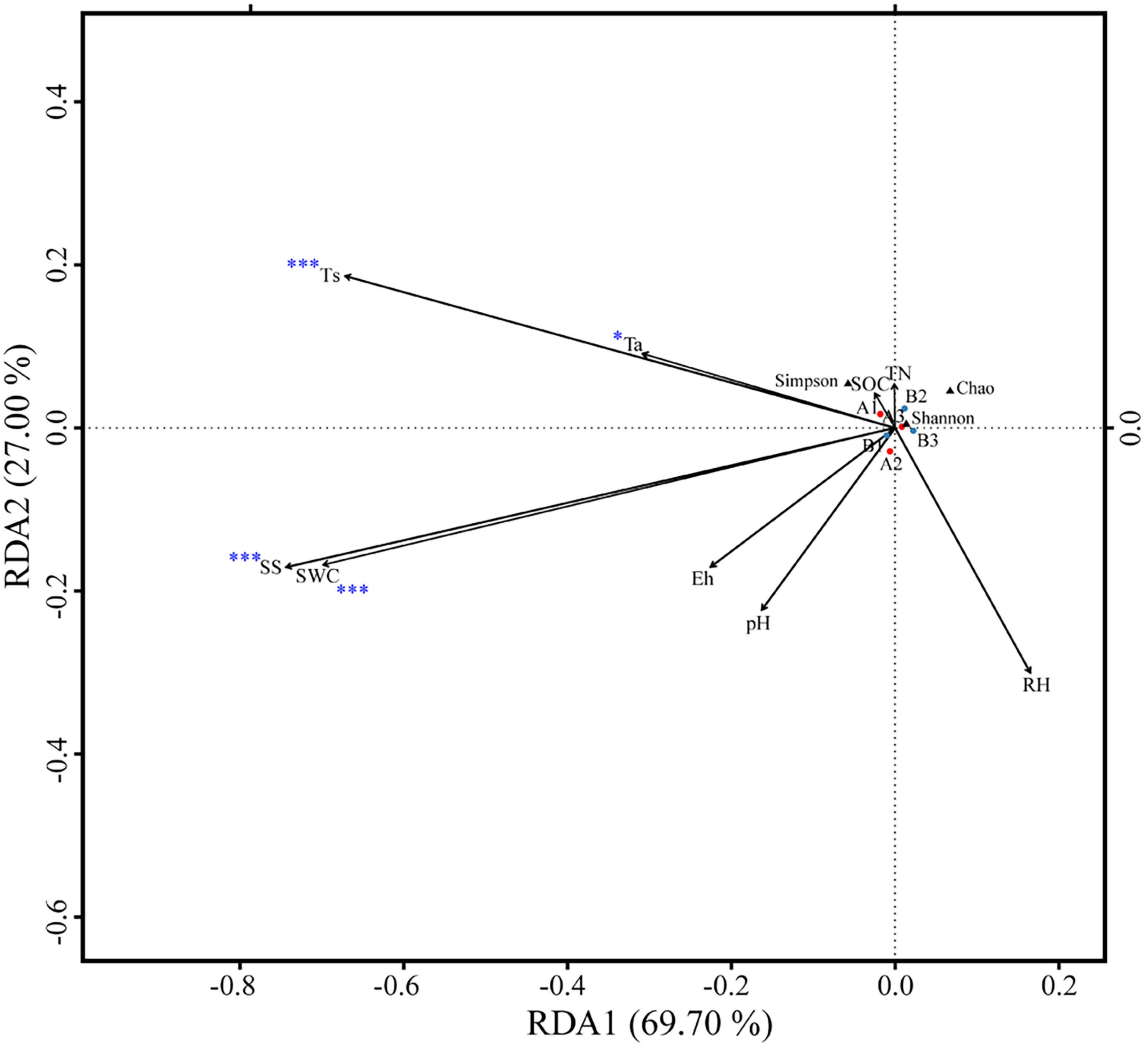
Redundancy analysis of soil bacterial alpha diversity as explained by environmental variables (*p < 0.05; and ***p < 0.001).

**Figure 6:**
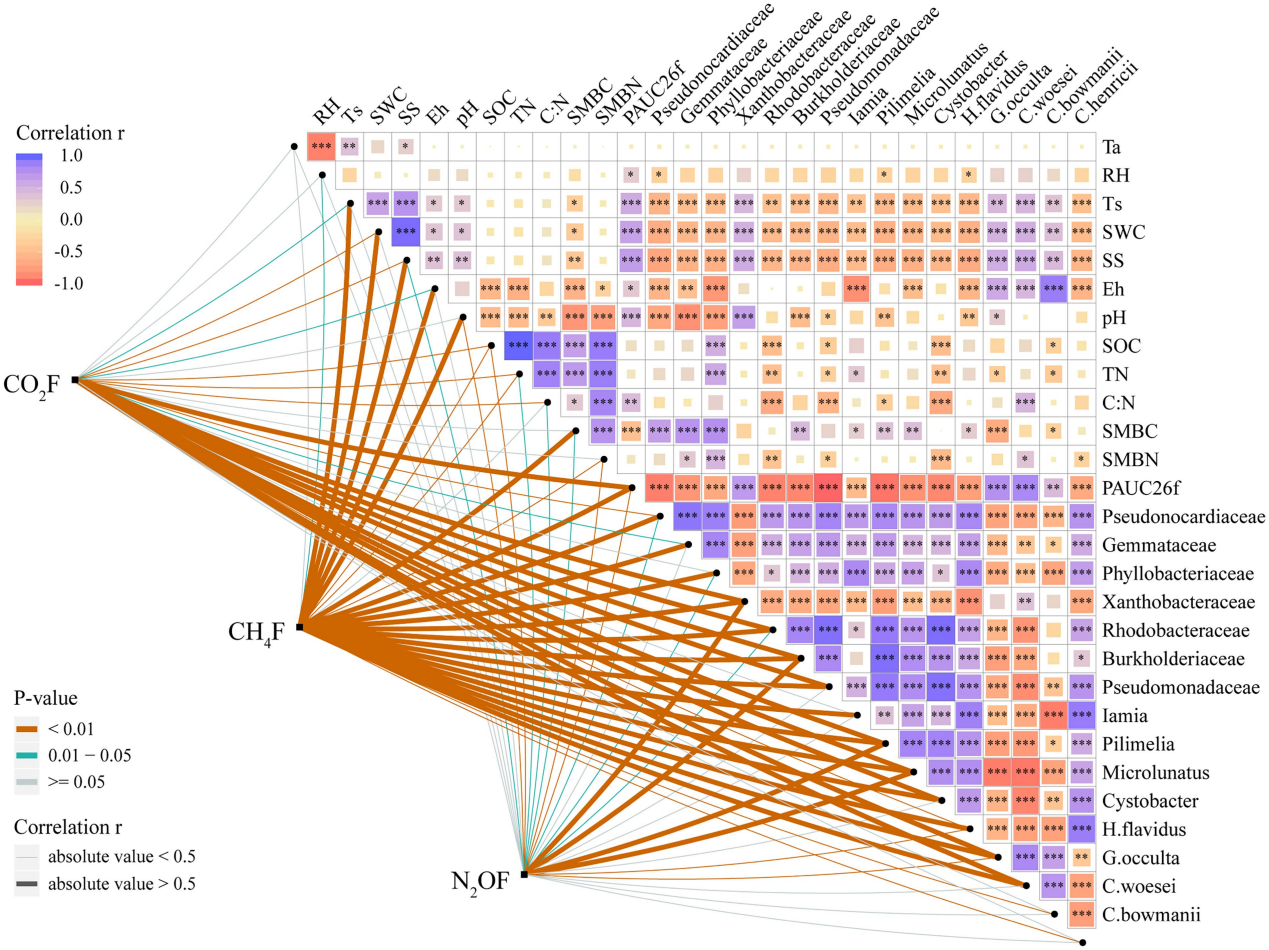
Pairwise comparisons of environmental variables with greenhouse gases. The color gradient indicates Pearson’s correlation coefficient. Each greenhouse gas is paired with each environmental variable by a partial Mantel test. Line width corresponds to Mantel’s r statistic for the corresponding distance correlations, and line color denotes the statistical significance based on 999 permutations (*p < 0.05; **p < 0.01; and ***p < 0.001).

OTUs numbers varied from 659 ± 25 to 687 ± 43 after two DFTCs. The principal coordinate analysis plot indicated that soil samples formed distinct clusters in the ordination space (Supplementary Figure S2), although the effect of DFTCs was not significant (*p* = 0.608). The relative abundances of 17 known bacteria at the different taxonomic levels, including eight families, four genera, and five species, showed significant changes after two DFTCs (Table 2). The relative abundances of *PAUC26f* and *Xanthobacteraceae* significantly decreased, but those of other families including *Burkholderiaceae*, *Gemmataceae*, *Phyllobacteriaceae*, *Pseudomonadaceae*, *Pseudonocardiaceae*, and *Rhodobacteraceae* distinctly increased (*p* < 0.05). Meanwhile, the relative abundances of *Cystobacter*, *Iamia*, *Microlunatus*, *Pilimelia*, *Caulobacter henricii*, and *Humicoccus flavidus* significantly increased, but that of *Clostridium bowmanii*, *Conexibacter woesei*, and *Gaiella occulta* distinctly decreased at the genus and species levels (*p* < 0.05). This finding indicated that bacteria of different families, genera, and species exhibited different responses to DFTCs. Moreover, the result indicated an increased trend toward alpha and beta diversities of bacteria from pre- to post-DFTCs (*P*_Chao_ = 0.288, *P*_Shannon_ = 0.508, *P*_Simpson_ = 0.842, and *P*_Beta_ = 0.114) (Figure 3). Redundancy analysis showed that the first and second axes explained 93.60% and 4.86% of the total variance in 17 known soil bacterial communities, respectively. The bacterial community composition was significantly correlated with Ta (*p* < 0.05), RH (*p* < 0.05), Ts (*p* < 0.001), SWC (*p* < 0.001), SS (*p* < 0.001), Eh (*p* < 0.01), and pH (*p* < 0.001) (Figure 4). The redundancy analysis of bacterial diversity and environmental variables showed that SS (*p* < 0.001), SWC (*p* < 0.01), Ts (*p* < 0.001), and Ta (*p* < 0.05) were the key environmental variables that significantly affected bacterial alpha diversity (Figure 5). In addition, the results of Pearson correlation analysis suggested that Ts, SWC, and SS were all significantly associated with 17 known bacterial abundances (*p* < 0.05) (Figure 6). In general, these results indicated that Ts, SWC, and SS were important variables affecting the composition and alpha diversity of soil bacterial communities.

**Table 2:** Differences in the relative abundance of soil bacteria at different taxonomic levels pre- and post-DFTCs.

Taxon	Abundances pre-DFTCs (SE)	Abundances post-DFTCs (SE)	Differ. (SE)	*p*-Value
**Family**				
*Burkholderiaceae*	0.000 (0.000)	0.095 (0.037)	0.095 (0.105)	0.043
*Gemmataceae*	0.041 (0.041)	0.189 (0.042)	0.148 (0.171)	0.045
*PAUC26f*	0.128 (0.010)	0.026 (0.014)	−0.102 (0.098)	0.005
*Phyllobacteriaceae*	0.038 (0.021)	0.283 (0.087)	0.245 (0.259)	0.038
*Pseudomonadaceae*	0.144 (0.054)	0.483 (0.062)	0.339 (0.337)	0.012
*Pseudonocardiaceae*	0.492 (0.064)	0.970 (0.108)	0.478 (0.512)	0.015
*Rhodobacteraceae*	0.286 (0.070)	0.729 (0.166)	0.443 (0.465)	0.048
*Xanthobacteraceae*	0.115 (0.037)	0.015 (0.015)	−0.100 (0.116)	0.047
**Genus**				
*Cystobacter*	0.113 (0.064)	0.500 (0.129)	0.387 (0.411)	0.031
*Iamia*	0.268 (0.037)	0.554 (0.100)	0.286 (0.299)	0.046
*Microlunatus*	0.491 (0.057)	0.764 (0.071)	0.273 (0.320)	0.020
*Pilimelia*	0.000 (0.000)	0.052 (0.014)	0.052 (0.055)	0.014
**Species**				
*Caulobacter henricii*	0.000 (0.000)	0.060 (0.019)	0.060 (0.066)	0.012
*Clostridium bowmanii*	0.124 (0.036)	0.054 (0.034)	−0.071 (0.071)	0.004
*Conexibacter woesei*	0.161 (0.047)	0.029 (0.029)	−0.132 (0.150)	0.037
*Gaiella occulta*	3.335 (0.550)	1.946 (0.148)	−1.389 (1.678)	0.036
*Humicoccus flavidus*	0.012 (0.012)	0.088 (0.005)	0.076 (0.078)	0.001

### Soil greenhouse gas fluxes changes and their influence variables

**Figure 7:**
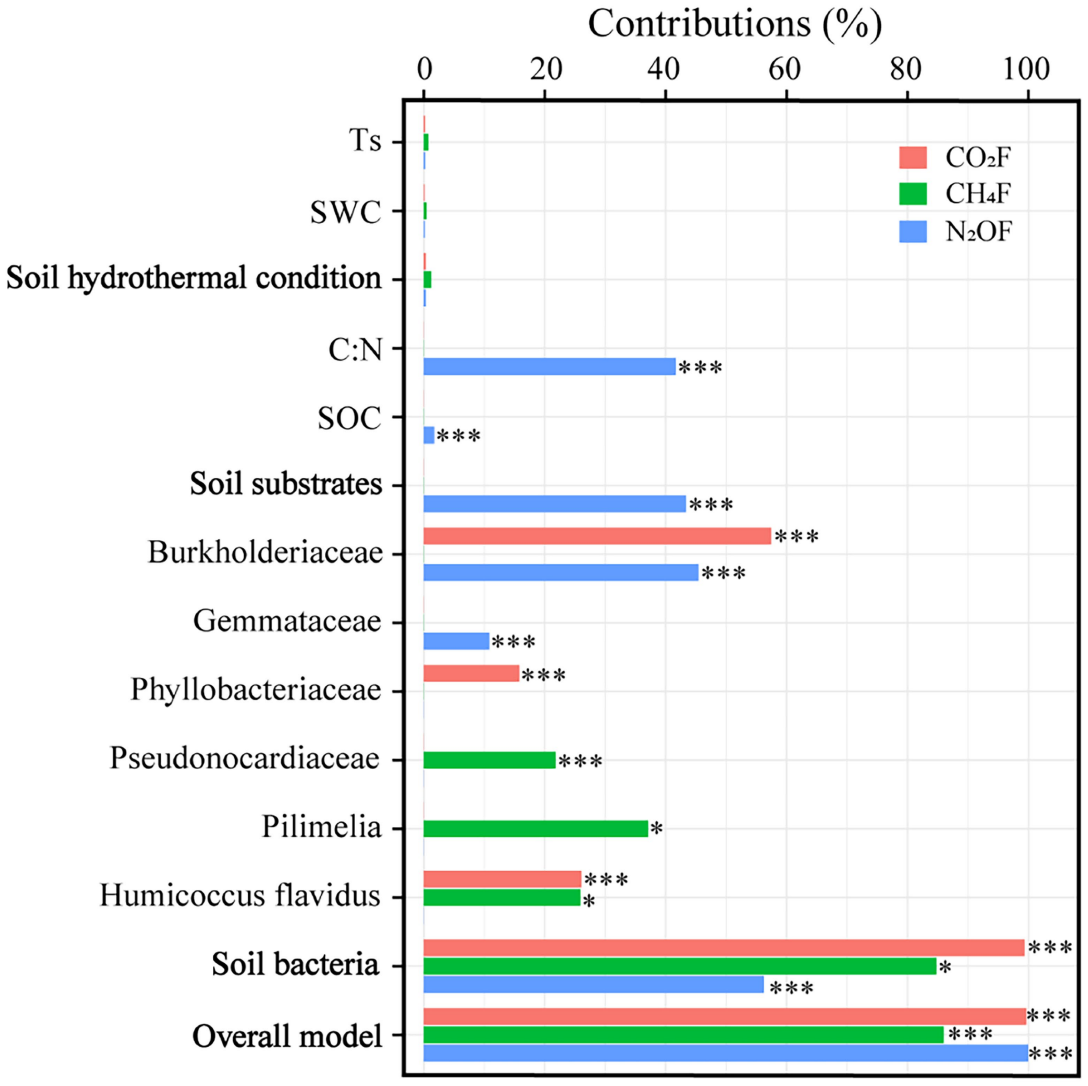
Contribution of environmental variables and soil bacteria to greenhouse gas fluxes (*p < 0.05; and ***p < 0.001).

The average fluxes of soil CO_2_, CH_4_, and N_2_O pre- and post-DFTCs in in-situ measurement were 91.531 mg·m^−2^·h^−1^, −0.024 mg·m^−2^·h^−1^, and 10.809 μg·m^−2^·h^−1^, respectively. After two DFTCs, diurnal mean CO_2_ flux significantly increased while CH_4_ flux significantly decreased, and N_2_O flux did not significantly change (Table 1). The results of the Mantel test showed that GHGs were more strongly correlated with soil bacteria than with environmental variables (Figure 6). Figure 7 showed that soil bacteria were the main driving variables affecting greenhouse gas fluxes, and their contribution rates to CO_2_, CH_4_, and N_2_O fluxes accounted for 99.68%, 98.59%, and 56.27% of the overall model, respectively. Among them, the soil bacteria that significantly contributed to CO_2_, CH_4_, and N_2_O fluxes were *Burkholderiaceae*, *Phyllobacteriaceae*,and *Humicoccus flavidus*; *Pseudonocardiaceae*, *Pilimelia*, *Humicoccus flavidus*; and *Burkholderiaceae*, *Gemmataceae*, respectively. Finally, soil substrates (C: N and SOC) contributed only to N_2_O flux.

## Discussion

Freeze–thaw cycles could alter soil hydrothermal conditions and physicochemical properties, and thus influence soil microbial activity, community structure, and diversity (Gao et al., 2022; Ji et al., 2022). As an engine of biogeochemical cycles (Falkowski et al., 2008; Sokol et al., 2022), soil microorganisms play a critical role in greenhouse gas emissions, which may have potentially positive climate feedback in the permafrost regions (Bardgett et al., 2008; Jansson and Hofmockel, 2020). Therefore, this study analyzed the effects of DFTCs on topsoil bacteria of the active layer and surface greenhouse gas fluxes by in-situ experiments in the permafrost regions of the Shule River headwaters on the western part of Qilian Mountains, northeast margin of the QTP. Our results demonstrated that the composition of soil bacteria in phyum level remained unchanged in the permafrost regions after two DFTCs, consisting mainly of *Proteobacteria*, *Acidobacteria*, *Actinobacteria*, *Chloroflexi*, and *Gemmatimonadetes* (Figure 2), which were in agreement with the results of previous studies (Zhang et al., 2014; Wu et al., 2022). This indicated that the same soil environment had similar bacterial community composition. Soil bacteria adapted to environmental disturbances after prolonged exposure to harsh and changing environments (Männistö et al., 2009). In addition, we observed no significant change in bacterial alpha and beta diversities after two DFTCs (Figure 3). It suggested that bacteria in the active layer permafrost soil could withstand the stress of freeze–thaw cycles. Consistent with some studies (Ji et al., 2022; Liu et al., 2022), freeze–thaw cycles did not change bacterial diversity. However, Li et al. (2022) found that freeze–thaw cycles significantly altered bacterial diversity. This might be due to the differences in freezing intensity, frequency, and SWC between in-situ experiment and laboratory simulation. Moreover, it might also be the result of different ecological strategies adopted by different microorganisms in various ecosystems for the freeze–thaw cycles (Männistö et al., 2018). Past studies reported that freeze–thaw cycles had different degrees of influence on soil microorganisms (Walker et al., 2006; Ren et al., 2018). We observed significant changes in soil bacterial abundance at the genus, family, and species levels after two DFTCs (Table 2), implying that autumn DFTCs altered soil bacterial communities, as reported by Ren et al. (2018). This was related to the adaptations of microbial taxa (Han et al., 2018; Liu et al., 2022). Due to various bacterial taxa having different adaptations, their relative abundance responded differently to the freeze–thaw cycles. Our results were in line with previous studies (Ade et al., 2018; Juan et al., 2018), which showed that Ts, SWC, and SS were the main environmental variables influencing soil bacteria (Figures 4, 6). Freeze–thaw cycles might lead to changes in water availability, as well as the redistribution of mineral elements and nutrients through fluctuation in Ts and repeated phase shifts of water, thus affecting soil microbial growth and metabolism (Zhang et al., 2016; Meisner et al., 2021; Liu et al., 2022).

In this study, CO_2_ flux increased significantly while CH_4_ flux decreased significantly, and N_2_O flux did not have a distinct change after two DFTCs (Table 1), indicating that DFTCs induced different effects on greenhouse gas fluxes (Wang et al., 2013; Gao et al., 2022). Previous studies have shown that the response of greenhouse gas emissions to freeze–thaw cycles was related to their patterns (*e.g*., frequency, duration and freezing temperature) (Wu et al., 2020; Liu et al., 2022). Changes in freeze–thaw patterns would affect soil hydrothermal conditions, physicochemical properties, and microbial communities (Yun et al., 2018; Ji et al., 2022), which would in turn affect greenhouse gas emissions. We found that soil bacteria were the main variables influencing greenhouse gas fluxes (Figure 7), while Ts, SWC, and SS were the main variables affecting soil bacteria (Figure 4). This result suggested that DFTCs changed soil environmental properties, affected the production or death of microbial communities, and consequently influenced the levels of GHGs emitted to the atmosphere. In addition, greenhouse gas emissions capacity might also be associated with the types and degradation degree of grassland (Guo et al., 2019; Wu et al., 2021). Soil respiration mainly included microbial and root respiration (Groffman et al., 2001; Wei et al., 2010). We found significant increases in CO_2_ flux and abundance of *Burkholderiaceae*, *Phyllobacteriaceae*, and *Humicoccus flavidus* after two DFTCs (Tables 1, 2), indicating an increase in bacterial activity related to CO_2_ (Sharma et al., 2006). Previous studies found that freeze–thaw cycles could lead to a sudden and brief increase in microbial respiration, which was associated with the release of nutrients from lysed cells (Schimel and Clein, 1996; Pesaro et al., 2003; Männistö et al., 2009). On the one hand, soil freezing might lead to microbial death and root decomposition during freeze–thaw cycles, providing readily available and easily degradable organic substrates for the surviving microorganisms after thawing, which allowed more contact between organic matter and microorganisms, and thus stimulating respiration (Larsen et al., 2002). On the other hand, freeze–thaw cycles could disrupt soil aggregate structure and release large amounts of active organic carbon for microbial utilization, thus increasing the carbon source required for respiration (Matzner and Borken, 2008). In addition, microbial respiration could be maintained at high levels during multiple all-day freeze–thaw cycles (Larsen et al., 2002), resulting in increased CO_2_ flux (Kumar et al., 2013; Jansson and Tas, 2014; Schostag et al., 2019), in agreement with the results of studies by Skogland et al. (1988), Sharma et al. (2006), and Kato et al. (2005). However, freeze–thaw cycles had a limited impact on soil CO_2_ emission. If the substrate available was gradually consumed by soil microorganisms with decreasing Ts, SWC, and increasing freeze–thaw frequency, it might lead to a decreasing trend of soil CO_2_ emission rate (Herrmann and Witter, 2002; Juan et al., 2018).

More than 80% of CH_4_ in the atmosphere comes from the activities of soil microorganisms (Zhang et al., 2015). It was reported that the metabolic activities of methanogens and methanotrophs jointly regulated the uptake and emission of soil CH_4_ during freeze–thaw cycles (Wang et al., 2022). Frequent freeze–thaw cycles resulted in high-dynamic changes of soil CH_4_ flux in the permafrost regions of the QTP (Song et al., 2015). For example, Yun et al. (2018) found that the alpine steppe, in the permafrost regions of the QTP, was a source of CH_4_ in spring and a CH_4_ sink in autumn. We observed that CH_4_ flux decreased significantly and showed a negative value after two DFTCs (Table 1). These results implied that CH_4_ uptake was greater than its emission. CH_4_ was generally generated by microbial-mediated decomposition of organic matter in anaerobic environment (Zhang et al., 2021). Ts and SWC were the main variables affecting the bacterial community during freeze–thaw cycles (Figure 4; Hao et al., 2016; Liu et al., 2016). Before two DFTCs occurred, the higher SWC and Ts provided an anaerobic environment for methanogens, which favored CH_4_ production. Because methanotrophs were more tolerant than methanogens to low temperatures (Dunfield et al., 1993), the former were more active than the latter organisms as Ts and SWC decreased in autumn, which would oxidize CH_4_ to CO_2_ (Zhang et al., 2015). In addition, soil freezing obstructed gas exchange between the soil and atmosphere during freeze–thaw cycles, resulting in weak uptake of CH_4_. Zhang et al. (2021) reported that Ts was the main variable affecting daily CH_4_ uptake during the non-growing season. In our study, it was the beginning of the non-growing season (October 1–6), and the alpine meadow of the QTP might be a sink for CH_4_ in autumn as temperature decreased and freeze–thaw cycles intensified.

Microbial activity and substrate availability limitation played an important role in N_2_O emission during freeze–thaw cycles (Röver et al., 1998; Cheng et al., 2016). Freeze–thaw cycles had a significant effect on soil N_2_O flux (Koponen et al., 2004; Wu et al., 2020), but we observed that N_2_O flux tended to increase after two DFTCs. Ts and SWC were considered to be the primary variables controlling N_2_O emissions during freeze–thaw cycles (Yang et al., 2003; Chen et al., 2018a). However, we found that the effects of Ts and SWC on N_2_O flux were not significant (Figure 7). This might be attributed to milder DFTCs occurring under natural conditions and microorganisms adapting to a low-temperature environment compared with that under laboratory conditions, which did not result in massive microbial death during freeze–thaw cycles (Schmidt et al., 2007). Because organic matter released by the dead microorganisms through cell lysis increased the effective soil nitrogen content, which then stimulated denitrification (Cheng et al., 2016). Denitrification was mainly limited by low temperature and SWC in the permafrost regions (Li et al., 2012). Wu et al. (2014) and Li et al. (2012) noted that the soil N_2_O emission was low at relatively low SWC levels (32% or <30%). Therefore, our results suggested that SWC was lower (26.27%–30.49%) and might be insufficient to trigger the N_2_O emission threshold during the thawing process. Furthermore, if soil substrates were insufficient or unavailable, N_2_O production would also be restricted (Risk et al., 2013). Of course, it was also possible that N_2_O was consumed by microorganisms in the permafrost, resulting in an insignificant increase (Dang et al., 2022). We agreed with Mafa-Attoye (2021) that *Burkholderiaceae* and *Gemmataceae* had impacts on N_2_O emission (Figure 7). Among them, *Burkholderiaceae* dominated the functional genes of NO_X_^−^ reduction process during anaerobic ammonia oxidation in the freeze–thaw cycle (Jia et al., 2021), and might consume N_2_O as a “sink” (Suenaga et al., 2021). This was the first in-situ experiment to explore the effect of autumn DFTCs on topsoil bacteria and surface greenhouse gas fluxes using a high-throughput pyrosequencing method in the permafrost regions of the Shule River headwaters on the western part of Qilian Mountains, northeast margin of the QTP. The laboratory experiments can be well controlled but cannot reproduce authentic soil freeze–thaw conditions. For example, most laboratory studies under controlled conditions did not consider plant, litter, and snow cover in their experimental design, and SWC, Ts and nutrient levels were usually adjusted (Nielsen et al., 2001; Henry, 2007; Wu et al., 2014, 2020). Therefore, our in-situ experiments provide the base to further validate and complement freeze–thaw cycle effects on microbial communities and greenhouse gas emissions. It is worth noting that we compared the changes in soil bacteria and GHGs between pre- and post-DFTCs; thus, our results need to be distinguished from other experimental results with control treatments without freeze–thaw cycles.

## Conclusion

This study explored the responses of topsoil bacteria in active layer and surface greenhouse gas fluxes to autumn DFTCs in the permafrost regions of the Shule River headwaters on the western part of Qilian Mountains, northeast margin of the QTP. Soil bacteria could be assigned into 35 phyla, 88 classes, 128 orders, 153 families, 176 genera, and 113 species. The dominant phyla were *Proteobacteria*, *Acidobacteria*, *Actinobacteria*, *Chloroflexi*, and *Gemmatimonadetes*. Our results reflected that soil bacterial communities and greenhouse gas fluxes exhibited varied response patterns to autumn DFTCs. After two DFTCs, 17 main soil bacterial communities significantly changed at the family, genus, and species levels, and bacterial diversity showed an increasing trend. CO_2_ flux increased significantly while CH_4_ flux decreased significantly, and N_2_O flux tended to increase after two DFTCs. Ts, SWC, and SS were the main variables affecting the composition and alpha diversity of soil bacterial communities during autumn DFTCs. Compared to soil hydrothermal condition and substrates, soil bacteria were the primary variables influencing greenhouse gas fluxes. This study focused on the effects of DFTCs on soil bacteria and GHGs, thus it is necessary to extend the study period and expand the study area to investigate the effects of freeze–thaw cycles on GHGs and microbial-mediated mechanisms at greater depths.

## Data availability statement

The datasets presented in this study can be found in online repositories. The names of the repository/repositories and accession number(s) can be found at: https://db.cngb.org/cnsa/, CNP0001094.

## Author contributions

SC contributed to conception and design of the study. ZL, YG, JC, and YJ performed the experiments and analyzed the data. SC and ZL wrote the paper. All authors contributed to the article and approved the submitted version.

## Funding

This project was supported by the National Natural Science Foundation of China (41871064), the National Key Research and Development Program of China (2019YFC0507404), the Qinghai Key R&D and Transformation Program (2020-SF-146), the Freedom Project of the State Key Laboratory of Cryospheric Science, Northwest Institute of Eco-Environment and Resources, Chinese Academy of Sciences (SKLCS-ZZ-2022), and the Qinghai Province High-level Innovative “Thousand Talents” Program.

## Conflict of interest

The authors declare that the research was conducted in the absence of any commercial or financial relationships that could be construed as a potential conflict of interest.

## Publisher’s note

All claims expressed in this article are solely those of the authors and do not necessarily represent those of their affiliated organizations, or those of the publisher, the editors and the reviewers. Any product that may be evaluated in this article, or claim that may be made by its manufacturer, is not guaranteed or endorsed by the publisher.
