# Supplementary material for: Effects of autumn diurnal freeze–thaw cycles on soil bacteria and greenhouse gases in the permafrost regions
Source: Front Microbiol. 2022 Dec 1;13:1056953. doi: 10.3389/fmicb.2022.1056953 (PMC9752937; doi:10.3389/fmicb.2022.1056953)
Supplement: Supplementary file 1 [file Data_Sheet_1.zip › Datasheet 1/Supplementary_Material.docx]

Supplementary Material


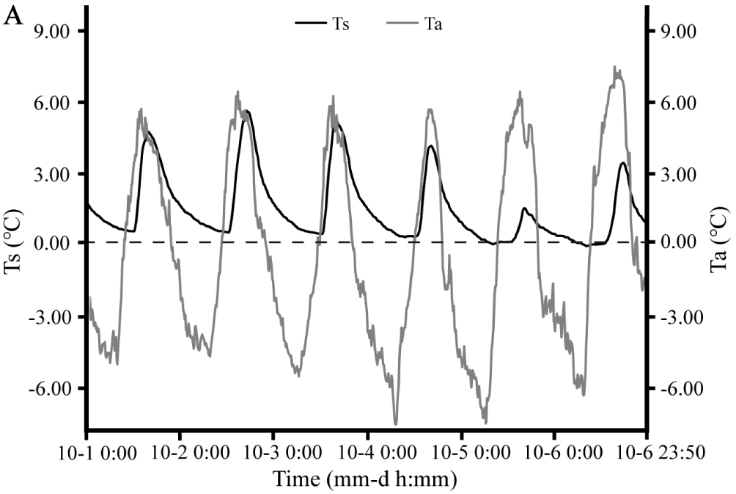


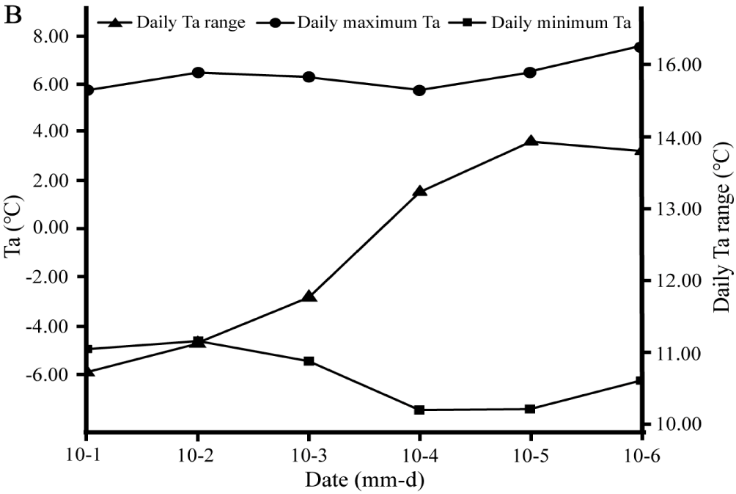


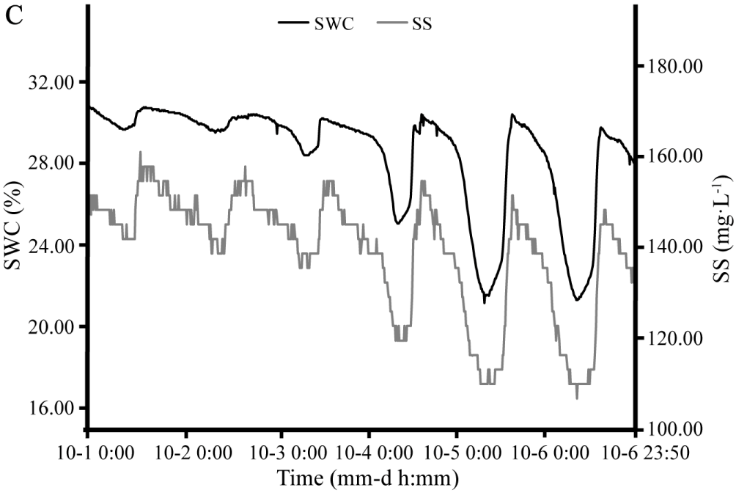


Figure S1. Daily ranges of soil hydrothermal conditions and salinity at 0-10 cm depth from October 1 to 6, 2011. Air temperature (Ta) and soil temperature (Ts) (**A**); Daily Ta range, daily maximum Ta, and daily minimum Ta (**B**); Soil water content (SWC) and soil salinity (SS) (**C**).


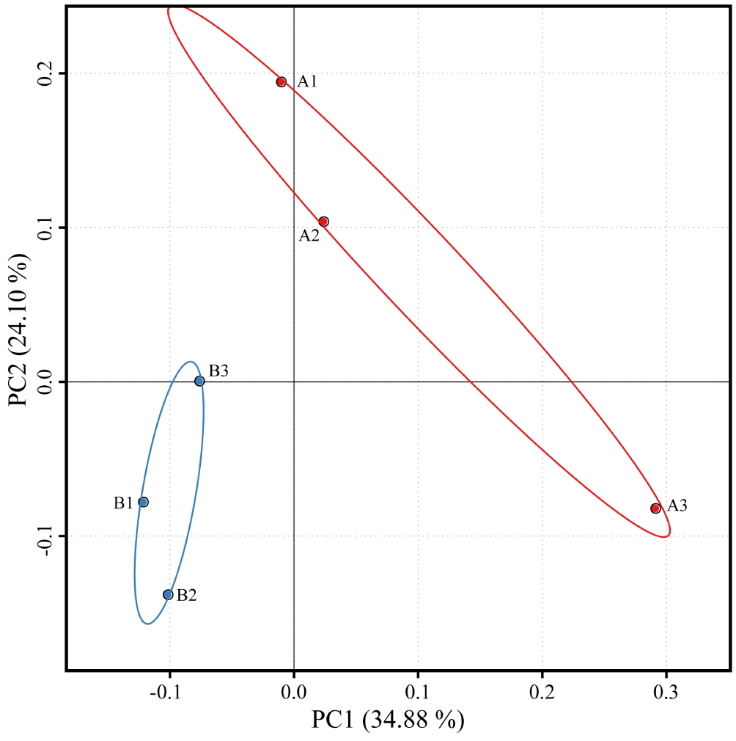


Figure S2. The principal coordinates analysis of differences in community composition of soil bacteria pre- and post-DFTCs.
