# Supplementary figures and images for: Effects of autumn diurnal freeze–thaw cycles on soil bacteria and greenhouse gases in the permafrost regions
Source: Front Microbiol. 2022 Dec 1;13:1056953. doi: 10.3389/fmicb.2022.1056953 (PMC9752937; doi:10.3389/fmicb.2022.1056953)

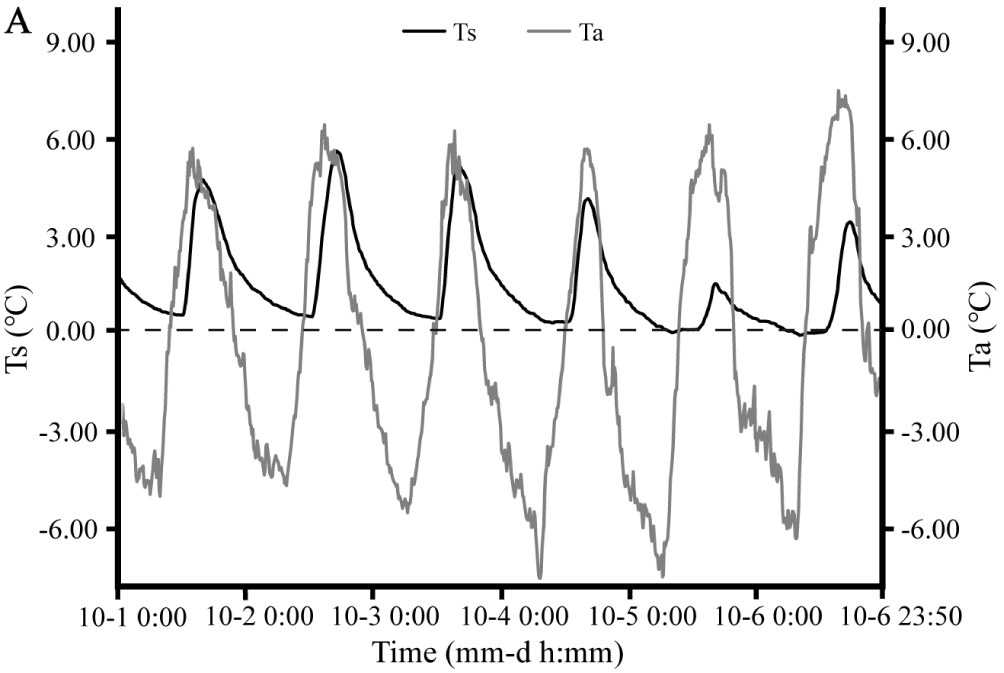

Supplement: Supplementary file 1 [file Data_Sheet_1.zip › Datasheet 1/Figure S1A.tif]

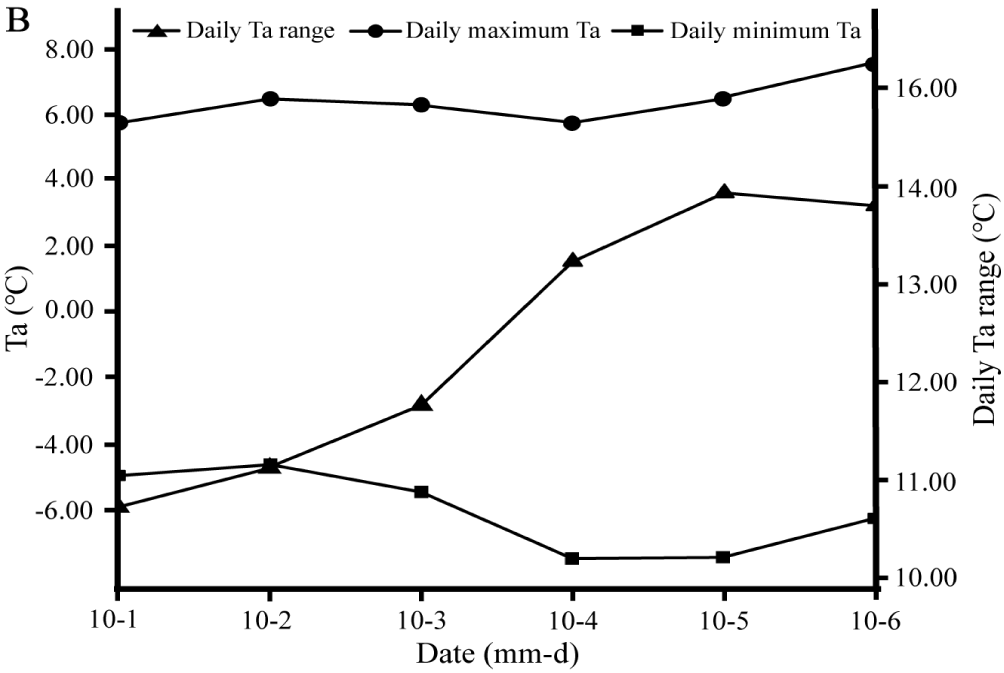

Supplement: Supplementary file 1 [file Data_Sheet_1.zip › Datasheet 1/Figure S1B.tif]

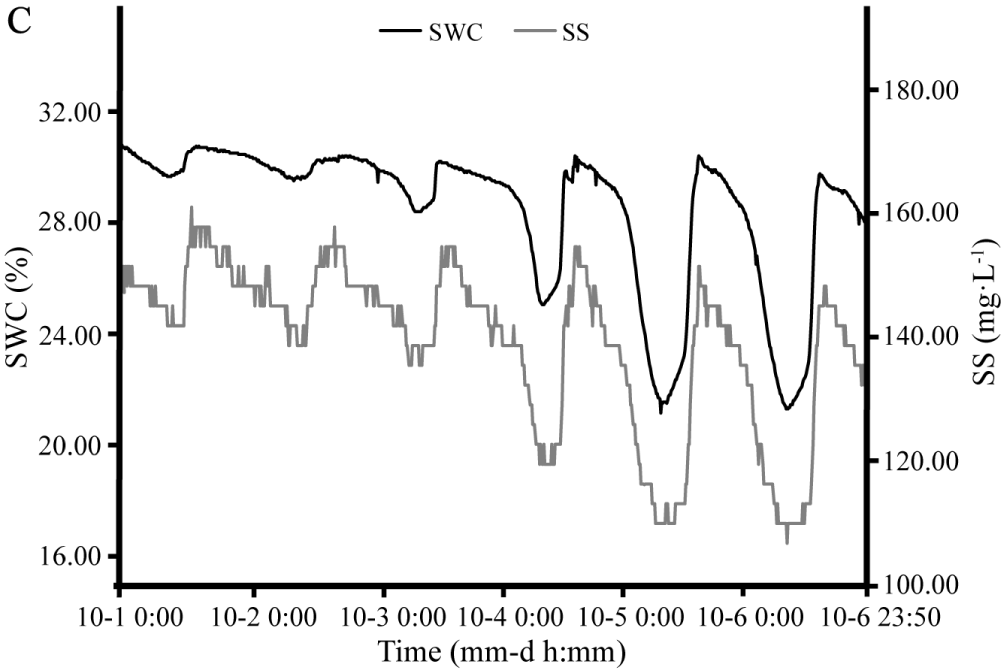

Supplement: Supplementary file 1 [file Data_Sheet_1.zip › Datasheet 1/Figure S1C.tif]

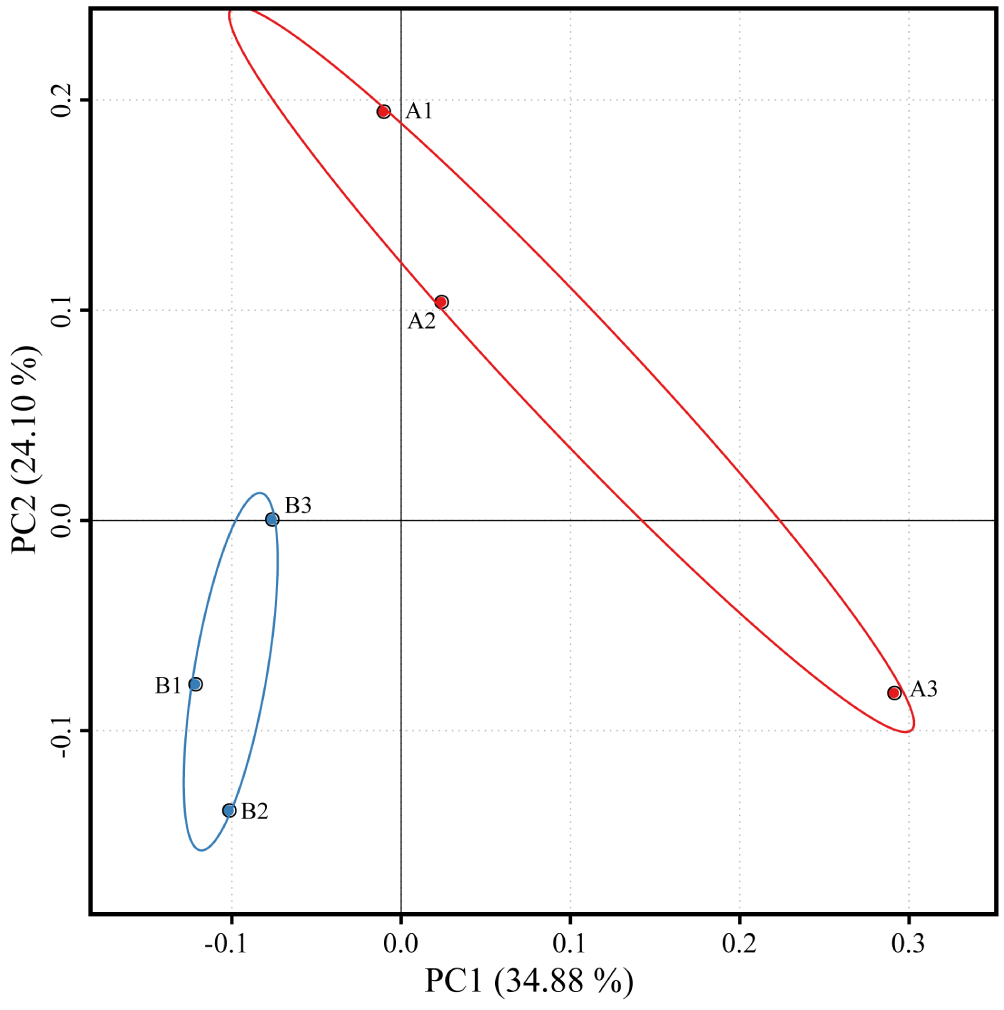

Supplement: Supplementary file 1 [file Data_Sheet_1.zip › Datasheet 1/Figure S2.tif]
